# A Comparative Study on the Efficacy of Intravenous Palonosetron Versus a Combination of Ondansetron and Dexamethasone as Prophylaxis for Prevention of Postoperative Nausea and Vomiting After Laparoscopic Surgeries

**DOI:** 10.7759/cureus.72214

**Published:** 2024-10-23

**Authors:** Deepak Krishnan, Arthi Asokan, Arunkumar Muthalu, Srinivasan Suganya, Chinthavali Sujatha

**Affiliations:** 1 Department of Anaesthesiology and Critical Care, Sri Venkateshwaraa Medical College Hospital and Research Centre, Puducherry, IND; 2 Department of Anaesthesiology and Critical Care, Sri Venkateshwaraa Medical College and Research Centre, Puducherry, IND; 3 Department of Critical Care, Mahatma Gandhi Medical College and Research Institute, Puducherry, IND

**Keywords:** dexamethasone, laparoscopic surgeries, ondansetron, palonosetron, ponv

## Abstract

Background: The most troublesome complaint after general anaesthesia and surgery, especially laparoscopic surgeries, is postoperative nausea and vomiting (PONV). We routinely use pharmacologic prophylaxis to prevent PONV. In patients undergoing laparoscopic procedures, we assessed the effectiveness of palonosetron compared to ondansetron combined with dexamethasone in preventing the incidence of PONV.

Methods and materials: This was a prospective, randomised, double-blind study that included 60 patients aged 18 to 60 years of either sex belonging to ASA physical status I or II undergoing elective laparoscopic surgeries. Before induction of anaesthesia, patients were randomised into two equal groups to receive either 0.075mg of palonosetron (group 1) or 4mg of ondansetron with 4mg of dexamethasone (group 2). Any incidence of nausea or vomiting along with the severity was assessed using the visual analogue scale, and the need for the rescue antiemetic was noted. Statistical analysis was done using an independent sample T-test, chi-square test, and Fisher’s exact test. P-value <0.05 was considered statistically significant.

Results: The overall incidence of PONV was 18% (11 patients), all of which were of mild to moderate severity. The palonosetron group had a lesser incidence of PONV, in three patients (10%) when compared to eight patients (26.6%) in the ondansetron and dexamethasone combination group over a period of 48 hours, but the difference was not statistically significant (P=0.854). The need for the rescue antiemetic was also comparable between both the groups (P=0.129), two patients required the rescue antiemetic (6.66%) in the palonosetron group, while in the ondansetron and dexamethasone group, six patients required the rescue antiemetic (20%).

Conclusion: Both palonosetron and ondansetron with dexamethasone prove to be comparably effective in preventing PONV in laparoscopic surgeries and achieving a complete response for a longer period, thus requiring fewer rescue medications with no adverse reaction.

## Introduction

In the postoperative period, the second most common complaint after pain is nausea and vomiting [1]. The causes of postoperative nausea and vomiting (PONV) are multifactorial and may be due to patient-related factors like female gender, nonsmoking individuals and previous history of PONV or motion sickness or anaesthesia-related factors like use of volatile agents and postoperative opioids [2]. In laparoscopic procedures, peritoneal distension caused by carbon dioxide insufflation may activate vagal afferents, promote vomiting by activating the vomiting centre and cause abdominal discomfort [3].

Although PONV usually lasts for a short period, it can cause serious side effects. These include dehydration, electrolyte imbalances, an increase in pain perception, and in rare cases, suture dehiscence and oesophageal rupture. PONV can also lead to feelings of depression and discontent and may delay discharge from the postoperative anaesthesia care unit in daycare surgeries [4].

Ondansetron is considered the gold standard antiemetic for the management of PONV [5]. However, it has a shorter half-life, requiring frequent doses. The combination of ondansetron and dexamethasone has been found to significantly reduce the incidence and intensity of PONV for a longer duration [6-9]. Palonosetron, a 5-HT3 receptor antagonist, is being researched for its potential in preventing PONV [10,11]. It has a superior safety profile and longer half-life compared to first-generation 5-HT3 receptor antagonists like ondansetron. However, there is a paucity of research comparing palonosetron to ondansetron and dexamethasone in terms of efficacy, especially in laparoscopic procedures.

This prospective randomised study was conducted to compare the efficacy of palonosetron versus the combination of ondansetron and dexamethasone in patients undergoing laparoscopic procedures to find the optimal antiemetic regimen for preventing PONV in laparoscopic procedures.

## Materials and methods

After obtaining ethical committee approval and registering this prospective, randomised controlled trial with the Clinical Trial Registry of India (CTRI/2022/10/046442), 60 individuals aged 18-60 years with physical status Class I or II as per the American Society of Anesthesiologists (ASA), scheduled for elective laparoscopic surgeries between November 2022 and May 2023, were recruited after obtaining written informed consent. Pregnant and lactating patients, obese individuals with BMI > 35 kg/m^2^, patients with a history of motion sickness, with any contraindication or allergy to the study drug, and those on steroid therapy, antiemetics, or on any medication known to cause nausea and vomiting were excluded.

A computer-generated block randomisation sequence was used to randomise the participants to either of the two groups, to receive either Inj. Palonosetron 0.075mg or Inj. Ondansetron 4mg with Inj. Dexamethasone 4mg, intravenously before induction of anaesthesia, and allocation was done using a serially numbered opaque sealed envelope technique. The drugs will be loaded and administered by an anaesthesiologist not involved in further study. The patients and the observer involved in data collection were blinded from the group allocation.

On the day before surgery, patients received standard premedications like Tab. Pantoprazole 40mg, Tab Metoclopramide 10mg, and Tab. Alprazolam 0.25mg according to the institute protocol, and they were explained about the Visual Analogue Scale (VAS) for nausea during preanesthetic assessment. On the day of surgery, vitals were checked, and ASA standard monitors were attached. Patients were premedicated with an injection of Midazolam 1mg i.v. and Inj. Glycopyrrolate 0.2mg i.v, and the study drug was administered just before induction of anaesthesia. Anaesthesia was induced with Inj. Fentanyl 2 μg/kg i.v., titrated dose of Inj. Thiopentone sodium 3- 5mg/kg i.v. and paralysed with Inj. Vecuronium 0.1 mg/kg i.v. to facilitate endotracheal intubation. After tracheal intubation, anaesthesia was maintained with isoflurane and oxygen (O_2_)- medical air mixture (50:50 ratio). Laparoscopic surgery was done with the pneumoperitoneum created with carbon dioxide (CO_2_) to an intra-abdominal pressure of 10-14 mm Hg.

In the postoperative period, patients were observed for the primary outcome, that is, the occurrence of PONV. The severity of nausea was assessed with VAS (marked on a 10 cm line representing, 0 - no nausea (complete response), 1-3 mild nausea, 4 - 6 moderate nausea, 7- 10 severe nausea) postoperatively at 30 minutes, 1 hour, 2, 4, 6, 8, 10, 12, 16, 20, 24, 36, and at 48 hours. Complete response (CR), which was defined as no incidence of PONV during the 48-hour observation period, was noted. Inj. metoclopramide 10 mg i.v. was used as a rescue antiemetic to treat nausea if VAS ≥ 4, or for any vomiting episode and repeated as necessary. We recorded the time to receive the first rescue antiemetic and the total doses required as secondary outcomes. Inj. Paracetamol 1 gm in 100 ml normal saline i.v was given to all patients in the postoperative period for postoperative analgesia.

Sample size

A sample size of 60 was determined based on a study comparing the incidence of PONV, between two groups, to estimate a mean difference in incidence of PONV of 16%, with a confidence interval of 95% and power of 75%, using statistical software OpenEpi version 3 [12].

Statistical analysis

Data was entered in Microsoft Excel (Microsoft Corporation, Redmond, USA) and analysis was done using IBM SPSS Statistics for Windows, Version 23 (Released 2015; IBM Corp., Armonk, New York, United States). Normally distributed continuous data were expressed in mean and standard deviation and analysed using an independent t-test. Categorical data was expressed as numbers and percentages, chi-square or Fisher’s exact test was used to analyse the significance and a P-value < 0.05 was considered statistically significant.

## Results

**Figure 1:**
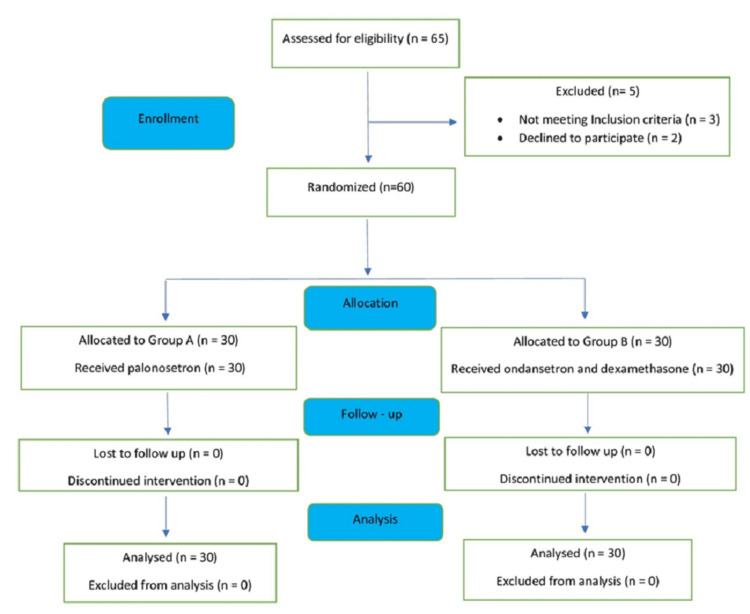
CONSORT diagram for sample allocation CONSORT: Consolidated Standards of Reporting Trials

 We enrolled 60 patients, all of whom completed the study with their data analysed as shown in the consort diagram (Figure 1).

The demographic variables like age, gender, BMI and ASA status of the patients between both the groups were comparable between the two groups (Table 1). The average duration of anaesthesia among group A was 107.0 (± 20.74) minutes and group B was 109.50 (± 21.87) minutes respectively. The difference in the duration was not statistically significant (p-0.651). The average duration of surgery among group A was 73.33 (±18.99) minutes and group B was 79.50 (±21.87) minutes respectively and the difference was not statistically significant (p - 0.248).

**Table 1:** Comparison of demographic characteristics between Group A and Group B P < 0.05 (Statistically significant). ASA: American Society of Anaesthesiology; SD: Standard Deviation; BMI: Body Mass Index; Group A: Palonosetron; Group B: ondansetron and dexamethasone. The duration of anaesthesia and duration of surgery were analysed using an independent T-test and p-value obtained

Variables	Group A	Group B	P-value
Age (mean± SD, years)	34.93 (±10.34)	34.80 (±11.33)	0.962
Gender (male/Female)	16/14	15/15	0.796
BMI (mean± SD,kg/m^2^)	25.9(±3.84)	25.9 (±3.46)	0.680
ASA (I/II)	22 / 8	23 / 7	0.766
Duration of general anaesthesia (mean± SD, hours)	107.00 (± 20.74)	109.50 (± 21.87)	0.651
Duration of surgery (mean± SD, hours)	730.33 (± 18.99)	79.50 (± 21.87)	0.248

The overall incidence of PONV was 11 patients (18%), all of which were of mild (VAS 1-3) to moderate (VAS 4-6) severity as shown in Table 2. The palonosetron group had a lesser incidence (3 (10%)) when compared to the ondansetron and dexamethasone combination group (8 (26.6%)) as shown in Table 2. There was no statistically significant difference between both the groups in the incidence of PONV during various timepoints over 48 hours (P=0.854).

**Table 2:** Incidence of PONV between the two groups (n=60) * P < 0.05(significant); Group A: Palonosetron; Group B: Ondansetron and dexamethasone; PONV: Postoperative nausea and vomiting. P-value based on Fischer's exact test.

Incidence of PONV	Group A (n=30)	Group B (n=30)	P-value
0 to 6 hours	1 (3.3%)	1 (3.3%)	1.000
6 to 12 hours	1 (3.3%)	3 (10%)	0.612
12 to 24 hours	0	2 (6.7%)	0.492
24 to 48 hours	1 (3.3%)	2 (6.7%)	0.500

Our secondary objective was to compare the requirement for rescue anti-emetic medications between the two groups (Table 3). Rescue antiemetic requirement was provided for 1 (3.3%) at six hours in each group. Two patients (6.6%) in group 2 required rescue analgesia at 12 hours and 24 hours; one (3.3 %) patient from each group required rescue analgesia at 48 hours.

**Table 3:** Rescue antiemetic requirement between groups (n=60) * P < 0.05 (significant); Group A: Palonosetron; Group B: Ondansetron and dexamethasone. P-value based on Fischer's exact test.

Rescue Antiemetic	Group A (n=30)	Group B (n=30)	P-value
0 to 6 hours	1 (3.3%)	1 (3.3%)	1.000
> 6 to 12 hours	0	2 (6.7%)	0.492
> 12 to 24 hours	0	2 (6.7%)	0.492
> 24 to 48 hours	1 (3.3%)	1 (3.3%)	1.000

**Figure 2:**
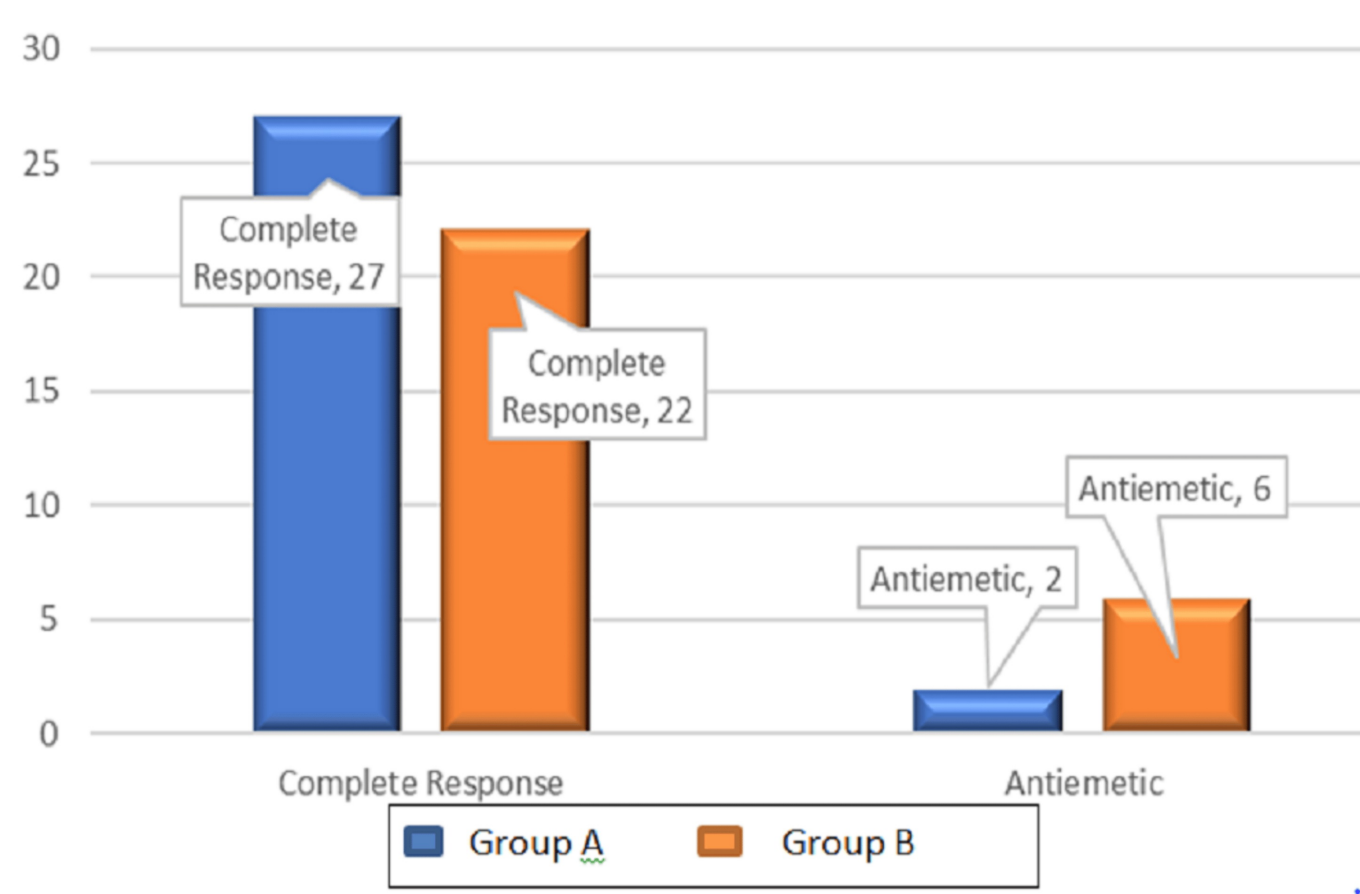
Bar diagram showing the incidence of the complete response and rescue antiemetic requirement between Groups A and B P-value = 0.129 for the rescue antiemetic and P-value = 0.095 for complete response. p <0.05: Statistically significant. Note: P-value based on the chi-square test.

In the palonosetron group, 27 patients showed CR, and two patients with PONV required rescue antiemetic (Figure 2). In the ondansetron and dexamethasone group, 22 patients showed CR, and six patients with PONV required rescue antiemetic (20%), though the former group required fewer rescue medications, this difference was statistically insignificant (P = 0.129 for the rescue antiemetic and P = 0.095 for complete response).

The comparison of the VAS for nausea between the two groups is shown in Table 4. In Group A, one patient reported mild nausea at 6-12 hours. Moderate nausea was reported among one patient at 0-6 hours and one patient at 24-48 hours. In Group B, one patient had mild nausea at 0-6 hours and one patient at 24-48 hours and moderate nausea was observed among one patient at 0-6 hours, two patients at 6-12 hours, two patients at 12-24 hours and one patient at 24- 48 hours. 

**Table 4:** VAS score between the two groups (n=60) Grading of nausea by the VAS score: VAS 0: No nausea (Complete response), VAS 1 to 3: Mild nausea, VAS 4 to 6: Moderate nausea, VAS 7 to 10: Severe nausea.

VAS Score	Group A (n=30)			Group B (n= 30)		
	Mild	Moderate	Severe	Mild	Moderate	Severe
0-6 Hours	0	1	0	0	1	0
6-12 Hours	1	0	0	1	2	0
12- 24 Hours	0	0	0	0	2	0
24-48 Hours	0	1	0	1	1	0

## Discussion

PONV affects 40-75% of those undergoing laparoscopic surgery under ambulatory anaesthesia and is a significant factor in determining enhanced recovery and early discharge from the hospital [13]. Factors such as age, gender, obesity, motion sickness, prior PONV, procedure length, choice of anaesthetic, opioid use, and pain can all impact PONV [14]. Avoiding the occurrence of PONV can decrease the risk of psychological distress, wound dehiscence, haemorrhage, increased intracranial pressure, and fluid and electrolyte imbalance. Recent advances in PONV prophylaxis include the use of pharmacological and non-pharmacological strategies, with a multimodal approach being the most effective. Ondansetron and dexamethasone are commonly used drugs for PONV prophylaxis, and palonosetron is an upcoming drug with better potency and efficacy. Palonosetron is a “second-generation” 5HT3 receptor antagonist, reported to be superior to the “first generation” 5HT3 receptor antagonists, because it binds at the allosteric site of the 5HT3 receptor and this binding may prevent attachment of 5HT at the orthosteric site of the receptor, explaining its long-lasting effects [15]. In our study, we compared the efficacy of palonosetron versus the combination of ondansetron and dexamethasone in preventing PONV in patients undergoing laparoscopic surgeries.

The results of our study showed that the palonosetron group had a lesser incidence of PONV when compared to the group of patients who received ondansetron plus dexamethasone over a period of 48 hours postoperatively but this difference was not statistically significant (P > 0.854). Our findings were similar to a prospective study done by Besra et al., on sixty patients who underwent laparoscopic cholecystectomy and received either palonosetron or ondansetron plus dexamethasone as PONV prophylaxis. They observed the incidence of PONV for 48 hours and found that the incidence of PONV at all time points was similar between the palonosetron group and ondansetron with dexamethasone group (P > 0.05), concluding both as equally potent [16]. Another study conducted by Dey et al. on laparoscopic gynaecological surgeries found a significantly lower incidence of PONV for 48 hours in the palonosetron group compared to the ondansetron and dexamethasone group (7.14% and 23.81%, P = 0.0488) [17]. Rajinikanth et al. also observed the incidence of PONV in laparoscopic cholecystectomy patients and concluded that both palonosetron-dexamethasone and ondansetron-dexamethasone combinations were equally effective in prophylaxis against PONV [18]. Park and Cho studied the use of ondansetron 8 mg and palonosetron 0.075 mg before anaesthesia induction in patients undergoing gynaecological laparoscopic surgeries. The incidence of PONV in the first 24 hours was significantly lower in the palonosetron group compared with the ondansetron group (42.2% vs 66.7%, respectively) and they concluded that palonosetron 0.075 mg was more effective than ondansetron 8 mg in preventing PONV [19]. There are few other studies with varied results; some show that both palonosetron and ondansetron are equally effective while others suggest palonosetron is superior to ondansetron and dexamethasone [20-25].

The number of patients with complete response was also observed in our study which is defined as no incidence of PONV during the 48 h observation period. Our results suggested that both the groups were comparable in achieving complete response with the incidence as follows: 27 patients in the case of Group A and 22 patients in Group B (P = 0.095 for complete response). Similarly, our results showed that the requirement for rescue antiemetic was also comparable between both the groups (P = 0.129 for rescue antiemetic). These findings of our study aligned with the study results by Rajnikant et al., done on laparoscopic cholecystectomy patients, which had demonstrated no significant difference in rescue antiemetic therapy requirement between patients receiving palonosetron with dexamethasone and patients receiving ondansetron with dexamethasone for a period of 0-48 hours. Their results showed that during the overall 0-48 h period, 9 patients (21.4%) in group palonosetron with dexamethasone compared to 12 patients (28.6%) in group ondansetron with dexamethasone required rescue antiemetic therapy and the difference between the groups was statistically not significant (P = 0.450) [18]. Prajapat et al. also showed no statistically significant difference in rescue antiemetic need between palonosetron with dexamethasone and ondansetron with dexamethasone groups (P > 0.05) [20]. They also observed that complete response (no PONV and no rescue antiemetic) was more in the palonosetron group compared with the ondansetron group and the need for rescue antiemetics was less during 0 - 48 h time interval (P>0.05). Another study by Dey et al., in laparoscopic gynaecological surgery showed that no rescue medication was required in the palonosetron group, whereas eight patients (19.05%) in the ondansetron with dexamethasone group required rescue antiemetic (P = 0.0093) [17]. There were no adverse effects reported in either group, suggesting both to be a safe choice in PONV prophylaxis.

Based on the observations from our study, both palonosetron and ondansetron with dexamethasone can be considered equally efficacious and safe in lowering the incidence and severity of PONV, with lesser rescue antiemetic requirements in the postoperative period. This suggests that palonosetron as a single drug is as potent for a longer duration, compared to combination therapy of ondansetron with dexamethasone.

This study has a few limitations. Firstly, palonosetron, a single drug was compared with the combination of two drugs ondansetron and dexamethasone. This was done since previous studies have already proven that palonosetron has a longer duration of action as an effective antiemetic and it has been used in laparoscopic surgeries as monotherapy to prevent PONV. Secondly, both the groups received the antiemetic drugs during induction of anaesthesia, although ondansetron is usually given 30 minutes prior to the end of surgery. This was done because the duration of laparoscopic procedures in our hospital is usually short lasting up to 1-2 hours and hence presuming it would not affect the outcome of our study. 

## Conclusions

We conclude that both palonosetron and ondansetron with dexamethasone can be an equally effective and safe choice for PONV prophylaxis in laparoscopic surgeries. Palanosetron as a single drug can be an effective alternative as it can achieve a complete response in more patients and hence can be used as monotherapy in preventing early and delayed PONV when compared with a combination of ondansetron and dexamethasone.
